# TransXLT: A novel ZTD prediction method with SASR-based data reconstruction

**DOI:** 10.1016/j.isci.2025.112328

**Published:** 2025-03-31

**Authors:** Shicheng Xie, Xuexiang Yu, Jiajia Yuan, Xu Yang, Mingfei Zhu, Yuchen Han, Min Wei, Zhongchen Guo

**Affiliations:** 1 School of Earth and Environment, Anhui University of Science & Technology, Huainan 232001, China; 2 Anhui Provincial Joint Laboratory of Urban 3D Real-Scene and Intelligent Security Monitoring, Huainan 232001, China; 3 School of Geomatics, Anhui University of Science & Technology, Huainan 232001, China; 4 School of Remote Sensing & Geomatics Engineering, Nanjing University of Information Science & Technology, Nanjing 210044, China; 5 School of Environment and Surveying Engineering Suzhou University, Suzhou 234099, China

**Keywords:** Applied sciences, Computer science, Navigation System

## Abstract

Traditional Zenith Tropospheric Delay (ZTD) models often face difficulties in maintaining prediction accuracy under complex meteorological conditions and data loss. To address this, we propose the transformer-xLSTM (TransXLT) model, which integrates spatial-temporal information from global navigation satellite system (GNSS) stations, ERA5 (global atmospheric reanalysis), and GPT3 (empirical ZTD estimation). Missing data are reconstructed using a sparse attention-based time series reconstruction (SASR) method. Experimental results show: (1) under a 120-h data loss, SASR reduces mean absolute error (MAE) by 24.5% compared to cubic Hermite interpolation; (2) SASR lowers training root mean square error (RMSE) by 15.1% versus direct data deletion; and (3) TransXLT achieves an average RMSE of 8.13 mm across six sites, reducing RMSE by up to 76.54% compared to benchmarks like CNN-LSTM and ERA5. Demonstrating robustness across varying latitudes, altitudes, and seasons, the model significantly advances ZTD estimation accuracy for GNSS applications.

## Highlights

• SASR imputes missing ZTD via masked prediction and interpolation
• TransXLT captures multi-scale temporal patterns for accurate ZTD forecast
• SHAP shows ERA5-ZTD and station height are key for ZTD prediction

## Graphical abstract

**Figure:**
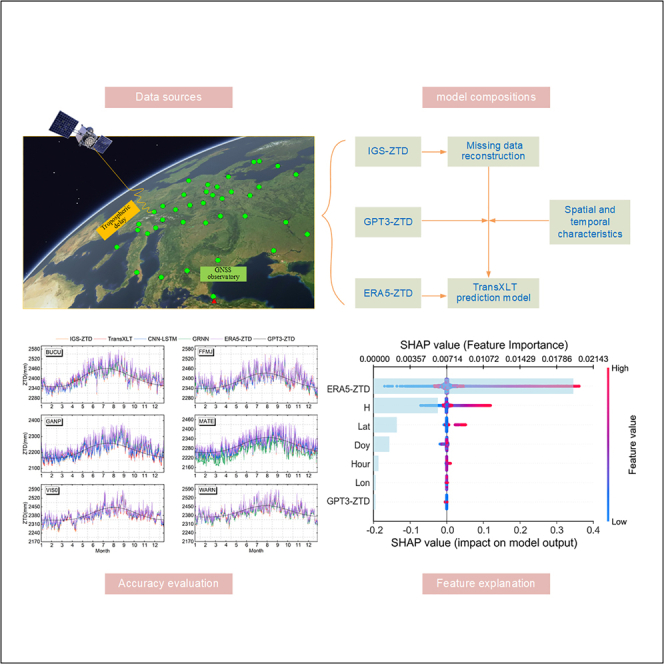

## Introduction

Tropospheric delay, caused by variations in atmospheric density and composition as electromagnetic signals pass through the troposphere (8–18 km above the Earth’s surface), is a major source of global navigation satellite system (GNSS) positioning errors, contributing inaccuracies of 2–20 m.^1^ Since directly measuring tropospheric delay along the signal path is challenging, it is typically estimated by applying a mapping function to the Zenith Tropospheric Delay (ZTD), which is composed of two components: Zenith Hydrostatic Delay (ZHD) and Zenith Wet Delay (ZWD).^2^ While ZHD accounts for approximately 90% of ZTD and can be accurately estimated using meteorological observations, ZWD, which is influenced by rapid fluctuations in atmospheric water vapor, poses greater challenges.^3^ Accurate ZTD modeling is therefore crucial to minimizing tropospheric delay-induced positioning errors.^4^

Tropospheric delay modeling has progressed through the development of both meteorological and non-meteorological models. Classical models, such as Saastamoinen,^5^ Hopfield,^6^ and Black,^7^ provide accurate ZTD estimates under stable conditions. However, these models show significant limitations in regions with high spatial variability and complex terrain.^8^^,^^9^ To address these challenges, non-meteorological models like the UNB series, developed by the University of New Brunswick, have been introduced.^10^^,^^11^ The UNB3m model, for example, achieves accuracy within 2 cm in North America but performs less effectively in high-altitude and low-latitude regions. Integrating machine learning techniques can improve its precision and adaptability.^12^^,^^13^ In addition, grid-based models, such as the global pressure and temperature (GPT) series and Vienna mapping function (VMF), utilize global meteorological reanalysis data to enhance ZTD estimation.^14^ VMF3 has shown superior performance over VMF1 across diverse terrains, though its effectiveness diminishes at higher altitudes, requiring further adjustments.^15^ Other models, such as GZTD/GZTD2,^16^^,^^17^ GTrop,^18^ GHop,^19^ IGGtrop,^20^ HGPT,^21^ and GGZTD-P,^22^ have been validated on regional and global scales, achieving accuracies of approximately 3–4 cm.

Machine learning has significantly advanced tropospheric delay modeling.^23^^,^^24^ Artificial neural networks (ANNs) have proven effective in capturing nonlinear relationships between meteorological variables and ZWD.^25^ Yin et al. developed a CNN-LSTM model for ZWD modeling in South America, achieving up to a 44% improvement in accuracy over the Saastamoinen model by employing three distinct modeling strategies.^26^ Similarly, Lu et al. introduced TropNet, a ConvLSTM-based model that incorporates spatiotemporal data from GOES-R satellites and the Global Forecast System (GFS). TropNet accurately predicts ZWD within 6 h, achieving RMS errors of 14.9 mm and 13.9 mm when compared with radiosondes and the VMF3 model.^27^

More recently, researchers have increasingly focused on transformer-based architectures, which excel at capturing long-term dependencies in time series modeling through parallelizable self-attention mechanisms. Zhang et al. validated the global-scale prediction accuracy of the transformer model using VMF-ZTD data from 505 stations of the Global Geodetic Observing System (GGOS), achieving a root mean square error (RMSE) of 1.8 cm.^28^ Hu et al. further improved computational efficiency by 17.8% with an informer-based model while maintaining equivalent accuracy.^29^ Furthermore, several variants of the transformer architecture, such as Autoformer,^30^ FEDformer,^31^ and iTransformer,^32^ shown superior performance compared to conventional machine learning approaches. Despite achieving notable success in various fields, Transformer models still encounter challenges in capturing short-term fluctuations and fine-grained temporal dynamics within ZTD modeling.

Although Long Short-Term Memory network (LSTM), one of the earliest deep learning models for time series, has been increasingly outperformed by transformer-based techniques due to their parallelizable self-attention mechanisms, recent advancements have significantly enhanced LSTM architecture, renewing research interest. The extended LSTM (xLSTM),^33^ incorporates exponential activation functions in the input and forget gates, along with normalization and stabilization techniques, improving storage flexibility and the handling of complex state-tracking tasks. Compared to conventional LSTM and Gated Recurrent Unit (GRU), xLSTM demonstrates greater flexibility and accuracy in capturing subtle temporal fluctuations, making it particularly effective for time series with rapid short-term changes. Furthermore, two novel memory structures have been introduced. The sLSTM model integrates multiple memory cells with a multi-head mechanism, enhancing time series prediction performance, while mLSTM employs matrix-based memory and covariance update rules to expand storage capacity. Notably, mLSTM eliminates the conventional memory mixing mechanism, enabling parallel training and significantly reducing training time.

Several studies have focused on developing high-completeness global ZTD datasets using large-scale training data to provide extensive GNSS station coverage worldwide.^34^^,^^35^ These datasets, characterized by vast data volumes and high completeness, demonstrate resilience to minor data loss, which has minimal impact on overall prediction accuracy. However, in practical applications, many regions suffer from limited GNSS station availability for ZTD retrieval. Stations in these areas often experience significant data loss due to network disruptions and environmental factors, leading to a shortage of reliable training data. Under such conditions, even small amounts of missing data can substantially degrade prediction performance. Some studies attempt to mitigate this issue by excluding stations with incomplete data, which further reduces the training dataset and impairs model performance.^24^^,^^36^ Alternatively, interpolation methods have been employed to fill data gaps, but these approaches often fail to handle the complexity and nonlinearity inherent in time series data.^37^^,^^38^^,^^39^ Additionally, have attempted to enhance ZTD prediction accuracy by integrating multiple models. However, most have focused on simple neural network structures, such as Backpropagation neural networks (BPNN) and Radial Basis Function (RBF) networks, which limit their ability to fully leverage the complementary strengths of different models and data sources.^40^^,^^41^^,^^42^

This paper tackles the challenges of ZTD data reconstruction and prediction using a two-stage approach. First, we propose the sparse attention-based time series reconstruction (SASR) method to restore missing ZTD data through masked prediction and interpolation, ensuring high-fidelity inputs for subsequent modeling. Second, we introduce the transformer-xLSTM (TransXLT) hybrid architecture, where the Transformer excels at capturing long-range dependencies and global features, while xLSTM is effective at modeling dynamic, nonlinear time series behaviors. ZTD time series exhibit both long-term variations (e.g., seasonal patterns) and short-term, localized wet delay fluctuations, requiring a balance between long-range and short-range dependencies. By combining the parallel processing capability of Transformer with the fine-grained memory updates of xLSTM, the model can effectively capture both global and local temporal features.

The TransXLT model integrates reconstructed ZTD data with spatiotemporal features (e.g., station coordinates and time indices) and external sources (e.g., GPT3-ZTD and ERA5-ZTD) to capture multiscale patterns. We also perform a comprehensive feature contribution analysis to evaluate the role of each input variable in prediction performance, addressing a gap in existing studies.

## Results and analysis

### Model parameter settings

The training process of deep learning models involves multiple hyperparameters, and their proper configuration is crucial for optimal performance.^43^ For the TransXLT model, key hyperparameters include epoch, batch size, time steps, encoding blocks, learning rate, dropout rate, fully connected layer dimension, and number of attention dimensions. Similarly, training the SASR model also depends on several important hyperparameters. Considering the dataset’s characteristics, we conducted a grid search during pre-training to identify the optimal configurations for all models. CNN-LSTM and General Regression neural network (GRNN) were selected as baseline models due to their established use in ZTD modeling.^4^^,^^44^^,^^45^ To ensure a fair comparison, the same input features, data splitting, and preprocessing procedures were applied across all models. The final hyperparameter settings are detailed in Table 1.

**Table 1:** Model parameter settings

Model	Parameters	Values	Parameters	Values
SASR	Epoch	1000	Batch size	32
	Number of transformer layers	4	Embedding dimension	64
	Feed-forward dimension	32	Number of heads	4
	Top-k	10	Dropout rate	0.1
TransXLT	Epoch	60	Batch size	32
	Time steps	10	Encoding blocks	3
	Learning rate	0.0005	Dropout rate	0.05
	Number of heads	4	Number of xLSTM Layers	2
	Fully connected layer dimension	16	Number of attention dimensions	32
CNN-LSTM	Epoch	60	Batch size	32
	Time steps	10	Learning rate	0.001
	Kernel Size	3	Number of filters	64
	Number of LSTM Layers	2	Dropout rate	0.05
GRNN	Bandwidth	0.035		

### Evaluation of reconstruction model accuracy

In this section, we evaluate the effectiveness of the SASR model in reconstructing missing data. As a commonly used interpolation method with low computational cost, the piecewise cubic Hermite interpolation (PCHIP) algorithm is often applied for the rapid restoration of relatively stable or low-frequency time series. Therefore, we use PCHIP as the baseline method to assess the performance of the SASR model.

To examine SASR’s performance under varying spatiotemporal conditions and data loss degrees, we selected four stations: AJAC (March), RIGA (June), PTBB (September), and SPT0 (December). In each station, continuous missing intervals of 24 h, 72 h, and 120 h were manually introduced in the middle of the time series for the respective month to simulate common ZTD observation interruptions. The missing data were then reconstructed using both the SASR and PCHIP methods. We compared the reconstructed outcomes with true values using bias and mean absolute error (MAE) metrics.

**Figure 1:**
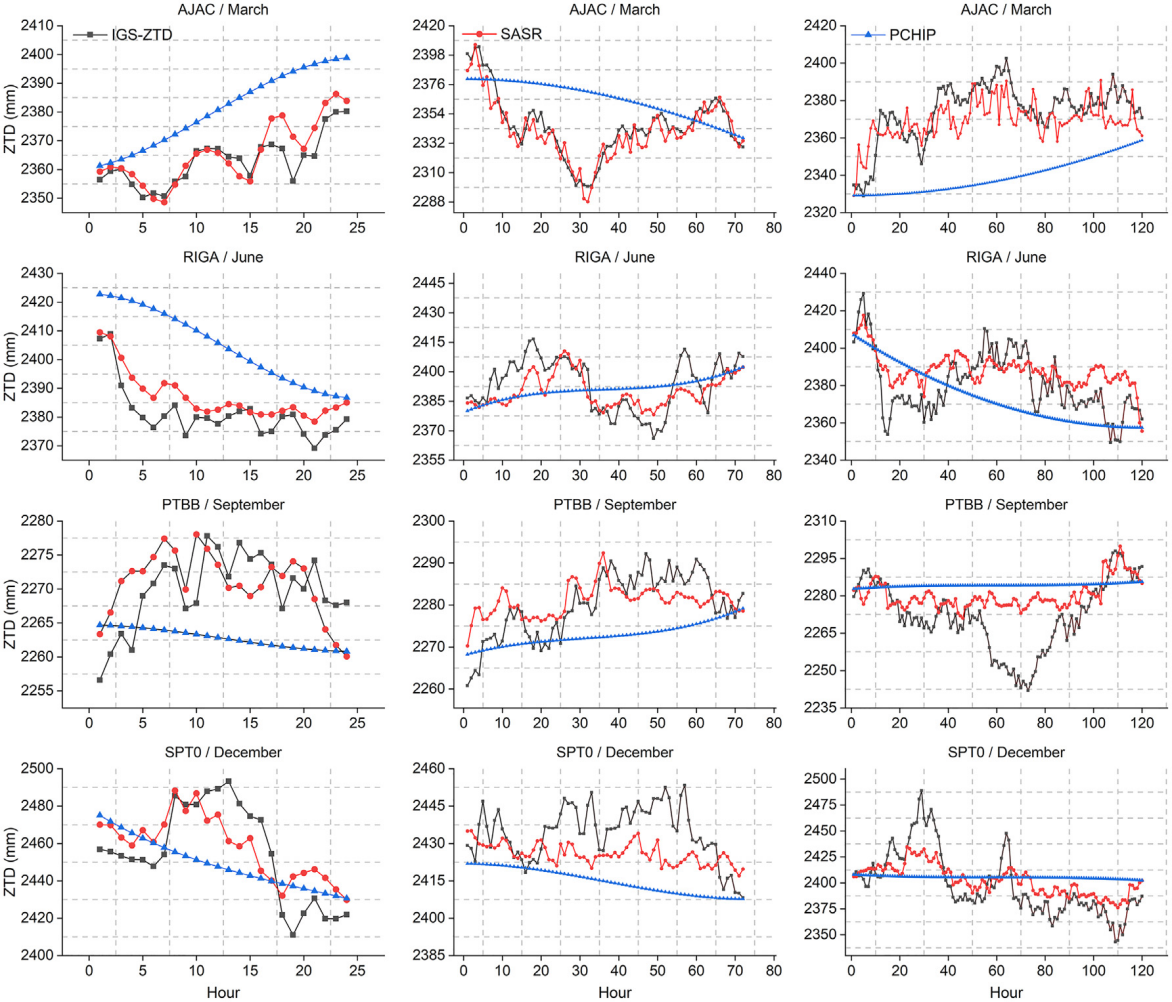
The reconstruction results for missing data using SASR and PCHIP are displayed The black curve denotes the true ZTD measurements, the red curve indicates SASR, and the blue curve signifies PCHIP. Data missing for 1 day (24 h), 3 days (72 h), and 5 days (120 h) are shown from left to right. The rows represent four stations across different months.

We present the data reconstruction results for various seasonal conditions and varying extents of missing data (Figure 1). Overall, SASR generally performs better in capturing fine-scale fluctuations for shorter missing periods (24 and 72 h), particularly during high-variability months like June or December. By contrast, PCHIP tends to produce smoother curves, which can be beneficial in more stable or low-frequency conditions (e.g., September) but can overlook rapid peaks and troughs in the data.

When the missing period extends to 120 h, both methods face greater challenges. Although SASR exhibits some lag effects and may not fully capture subtle variations, it generally achieves lower errors than PCHIP, particularly in scenarios with significant fluctuations. In contrast, PCHIP’s inherent smoothing effect, while easy to implement, suppresses sharp changes, resulting in higher bias and MAE values for high-frequency variations.

Table 2 quantitatively supports these observations. For shorter gaps (24 and 72 h), SASR consistently achieves lower bias and MAE than PCHIP. For example, at the AJAC station in March, SASR reduces bias and MAE by 86.1% and 77.4%, respectively, compared to PCHIP for a 24-h data gap, highlighting its strong ability to restore detailed variability. Even for extended gaps of 120 h during high-variability periods, such as June at the RIGA station, SASR maintains a 24.5% lower MAE than PCHIP, demonstrating superior robustness in capturing trends. However, PCHIP performs adequately for smoother ZTD patterns, such as the relatively stable periods in September, where its simpler approach yields comparable or only marginally higher errors.

**Table 2:** Accuracy comparison of data reconstruction methods (mm)

Missing Epoch/Hour		24		72		120	
Site/Month	Methods	Bias	MAE	Bias	MAE	Bias	MAE
AJAC/March	SASR	2.52	4.11	−3.81	6.99	−5.02	10.54
	PCHIP	18.19	18.19	19.68	26.23	−34.78	34.79
RIGA/June	SASR	5.99	6.16	−3.35	8.06	7.66	12.43
	PCHIP	23.53	23.53	−2.45	11.89	−6.56	16.45
PTBB/September	SASR	0.95	4.86	1.12	5.07	8.50	10.35
	PCHIP	−7.06	8.47	−7.06	7.96	12.5	14.95
SPT0/December	SASR	3.42	15.15	−8.52	11.73	4.31	16.71
	PCHIP	−5.98	21.46	−19.54	19.59	7.15	25.83

In summary, while PCHIP serves as a simple and effective baseline for stable or low-frequency data, SASR exhibits superior adaptability to significant fluctuations, making it particularly suitable for complex-frequency ZTD data across various seasons. As a result, SASR provides more reliable recovery of short-term missing values and enhanced robustness in high-variability scenarios, delivering higher-quality inputs for subsequent ZTD modeling tasks.

### The impact of missing data on modeling accuracy

To emphasize the importance of data integrity in TransXLT model training, we compared three preprocessing strategies for handling missing IGS-ZTD data from 33 stations: SASR reconstruction, PCHIP reconstruction, and directly using the original IGS-ZTD data with missing values. While directly using data with missing values may be considered a simple approach in some GNSS applications, it is generally regarded as suboptimal, as it reduces the amount of valid data and degrades model performance. Therefore, we included this method as a baseline to quantify the impact of leaving missing data unreconstructed.

**Figure 2:**
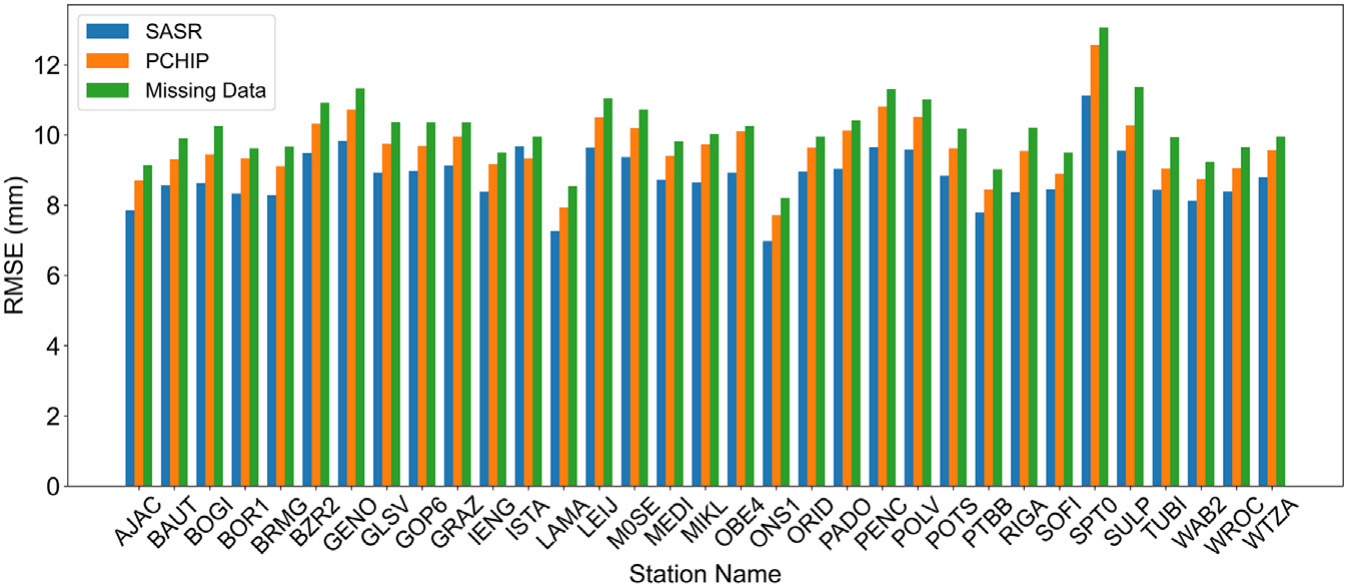
Accuracy of the model under different data processing strategies The blue and orange bands represent the training errors of the TransXLT model after recovering missing data using the SASR and PCHIP methods, respectively, while the green band denotes the training error when the model is trained directly on the original samples with missing data.

The influence of preprocessing strategies on model performance was evaluated by training the TransXLT-ZTD model on datasets processed by these methods and calculating the RMSE between the predictions and original IGS-ZTD values. As shown in Figure 2, using incomplete data disrupted spatiotemporal consistency and led to higher errors, with an average RMSE of 10.14 mm–15.1% higher than the RMSE obtained from SASR-reconstructed data. Both SASR and PCHIP reconstructions improved data consistency, with SASR achieving an average RMSE of 8.81 mm, 9.1% better than PCHIP. These findings emphasize the critical role of data integrity in accurate ZTD modeling. Although using missing data as a baseline is straightforward, it discards valuable associated features, whereas the SASR method effectively preserves both the quantity and quality of the data, resulting in more reliable predictions.

### TransXLT model accuracy evaluation

After reconstructing missing values, we obtained complete IGS-ZTD datasets for 33 stations spanning day 001 to day 365 of 2022. Input features include the Doy, hour, station latitude, longitude, ellipsoidal height, GPT3-ZTD and ERA5-ZTD with the output being the IGS-ZTD. At this stage, data normalization was applied, and the model was trained using the Adam optimizer and the mean squared error (MSE) loss function. Six stations were selected for final evaluation, with their details listed in Table 3.

**Table 3:** Test station information

Station	Lat (°)	Lon (°)	H (m)
BUCU	44.46394	26.12574	143.1376
FFMJ	50.09058	8.66497	178.2687
GANP	49.03471	20.32294	746.0258
MATE	40.64913	16.70446	535.6538
VIS0	57.65387	18.36732	79.75975
WARN	54.16979	12.10143	50.74287

**Figure 3:**
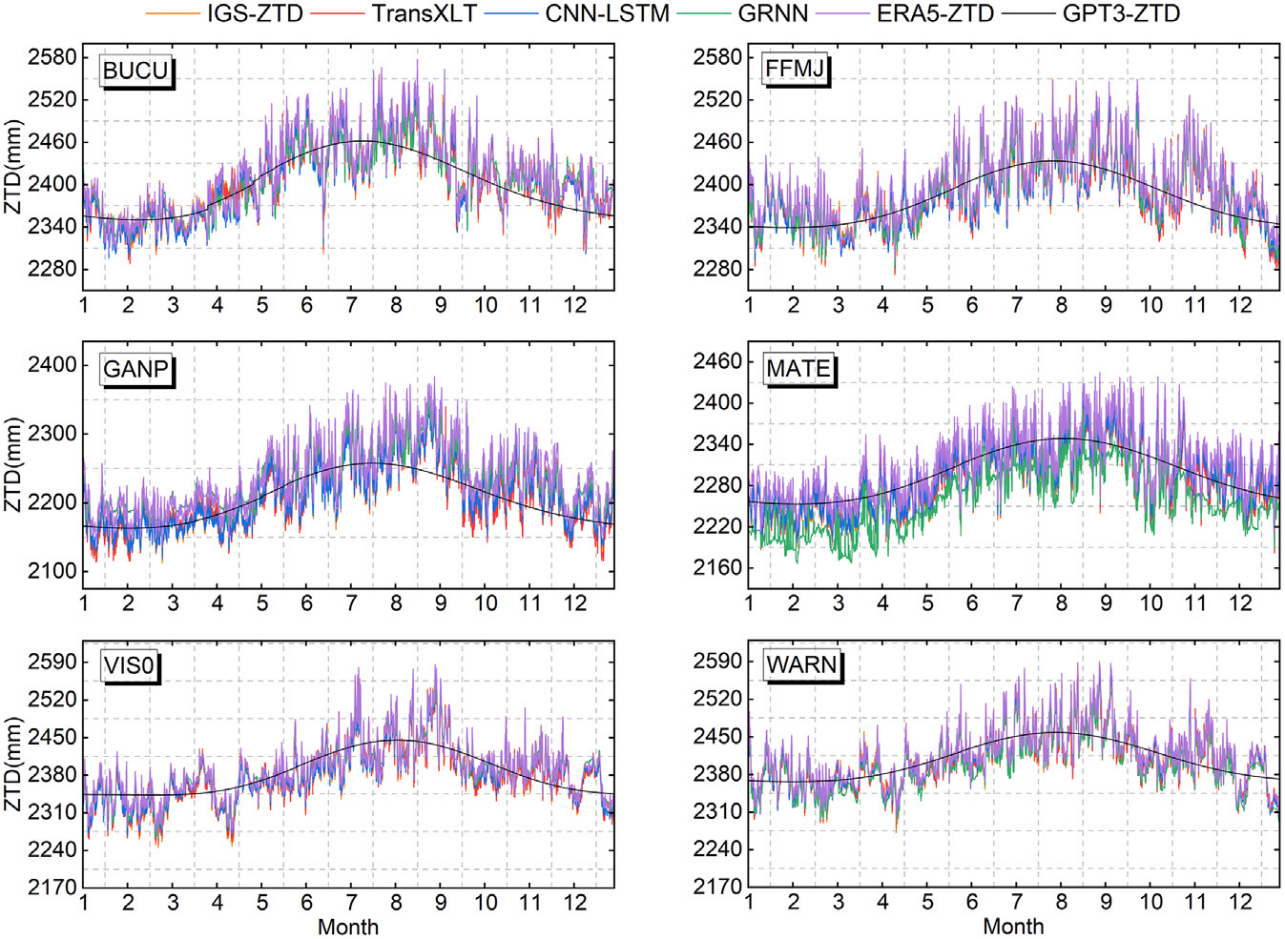
Comparison of ZTD results from different models ZTD values for 12 months were extracted from five models (TransXLT, CNN-LSTM, GRNN, ERA5, and GPT3) and compared with the ZTD product published by IGS.

We compare the ZTD predictions from TransXLT, CNN-LSTM, and GRNN with those calculated by the ERA5 and GPT3 models (Figure 3). Both TransXLT and CNN-LSTM models accurately capture ZTD fluctuation trends, particularly monthly variations in the annual cycle, aligning closely with IGS-ZTD. In contrast, GRNN shows notable deviations in certain periods, especially in months with large annual fluctuations (e.g., June and December), with significantly increased prediction errors. Although GPT3 reflects the overall annual ZTD variation trend, it exhibits considerable local deviations from IGS-ZTD.

**Figure 4:**
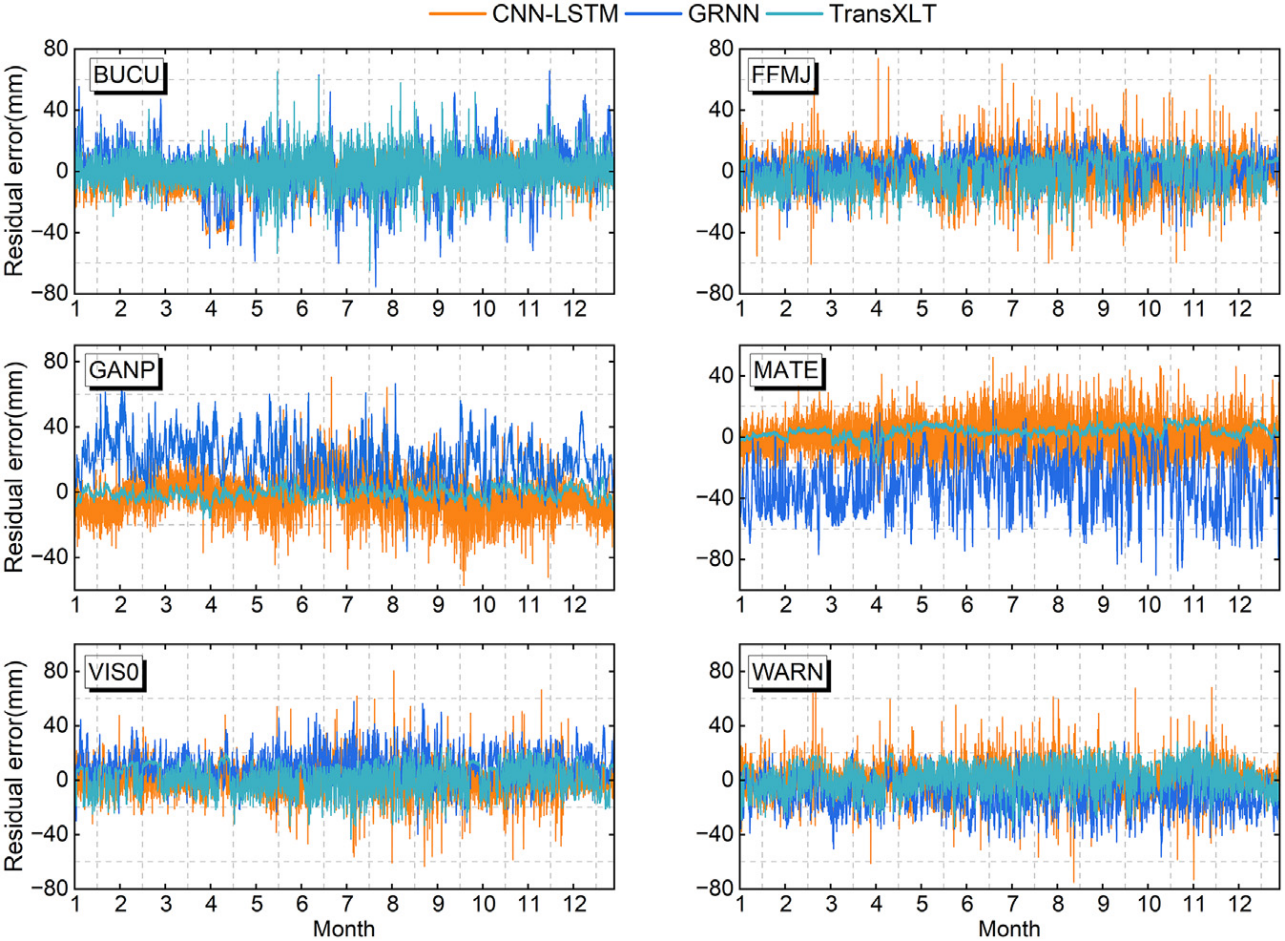
Residuals of ZTD predictions from the three models compared to IGS-ZTD Residual errors (mm) from CNN-LSTM (orange), GRNN (blue), and TransXLT (green) relative to IGS-ZTD at six test stations over a 12-month period. The horizontal axis indicates the month, and the vertical axis shows the residual magnitude.

We present the residual distributions of TransXLT, CNN-LSTM, and GRNN predictions relative to IGS-ZTD (Figure 4). TransXLT consistently achieves smaller, less volatile residuals across all stations, showing superior stability. At GANP and MATE stations, TransXLT residuals mostly fall within ± 10 mm, while CNN-LSTM and GRNN show more scattered residuals, some exceeding ±40 mm. Overall, TransXLT excels in high-volatility regions (e.g., BUCU and MATE) and high-variability seasons (e.g., summer and winter). This is due to TransXLT’s superior feature learning capabilities, enabling it to capture rapidly changing nonlinear atmospheric characteristics. Conversely, CNN-LSTM and GRNN perform slightly better in low-volatility stations (e.g., VIS0), excelling with stable data but struggling with complex changes.

**Figure 5:**
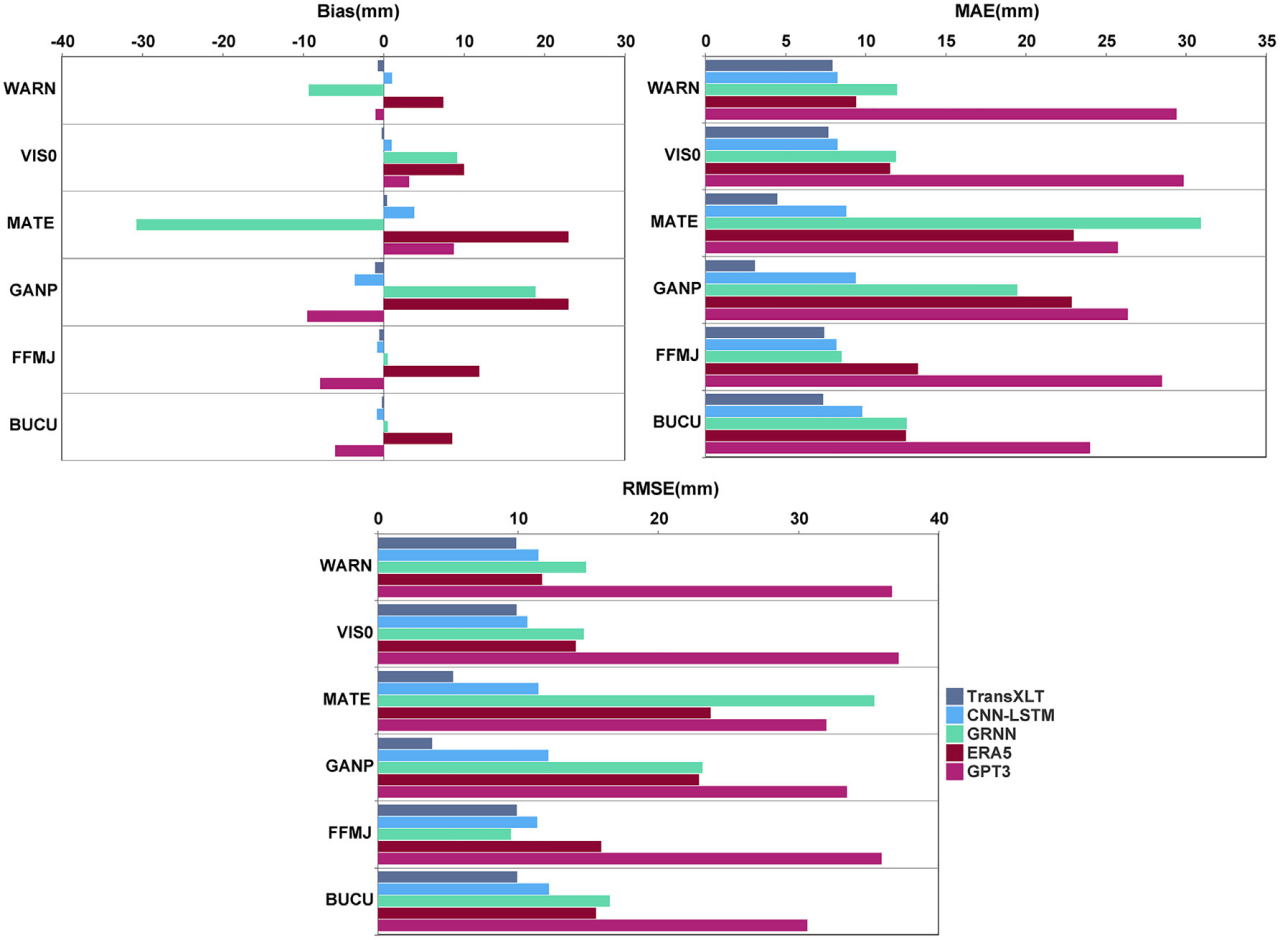
Bias, MAE, and RMSE metrics for different models at six test stations For details on how the error metrics were calculated, please refer to the quantification and statistical analysis section.

We present the TransXLT model’s stable and accurate predictions at all sites, reflected in consistently low Bias, MAE, and RMSE values (Figure 5; Table 4). At the BUCU site, TransXLT achieved a bias of −0.17 mm, while the other four models had an average Bias exceeding 1.5 mm, with ERA5 showing a bias as high as 13.97 mm. At high-variability sites, such as MATE and GANP, TransXLT’s RMSE is 5.36 mm and 3.86 mm, significantly outperforming GRNN’s 23.73 mm and 23.15 mm.

**Table 4:** Accuracy of five models evaluated across six test stations (mm)

Station	TransXLT	CNN-LSTM	GRNN	ERA5	GPT3
–	(Bias/MAE/RMSE)	(Bias/MAE/RMSE)	(Bias/MAE/RMSE)	(Bias/MAE/RMSE)	(Bias/MAE/RMSE)
BUCU	−0.17/7.34/9.93	−0.81/9.78/12.20	0.51/12.56/16.53	8.55/12.51/15.56	−6.02/24.00/30.63
FFMJ	−0.51/7.41/9.90	−0.78/8.17/11.35	0.52/8.49/9.48	11.89/13.26/15.92	−7.87/28.50/35.94
GANP	−1.02/3.08/3.86	−3.55/9.38/12.15	18.88/19.46/23.15	22.99/22.87/22.91	−9.47/26.37/33.47
MATE	0.40/4.48/5.36	3.81/8.78/11.44	−30.69/30.92/35.42	22.99/22.98/23.73	8.72/25.74/31.99
VIS0	−0.22/7.67/9.89	1.01/8.24/10.66	9.16/11.89/14.68	10.00/11.53/14.10	3.18/29.85/37.15
WARN	−0.70/7.92/9.86	1.06/8.24/11.44	−9.30/11.96/14.84	7.44/9.40/11.71	−0.98/29.41/36.68

Additionally, the CNN-LSTM model demonstrates robustness and relatively low errors at some sites. At the WARN site, CNN-LSTM achieves an RMSE of 11.44 mm, much lower than GPT3’s 36.68 mm. This suggests CNN-LSTM adapts well to medium variability data. However, at high-variability sites (e.g., MATE), its MAE and RMSE remain slightly inferior to those of the TransXLT model. CNN-LSTM’s capacity to model complex nonlinear relationships is evident, but its limited ability to handle extreme values slightly reduces accuracy for data with complex seasonal variations.

In contrast, the GRNN model exhibits significant bias in modeling ZTD under complex conditions, particularly at stations with large fluctuations in water vapor content, where it is more susceptible to climate variations. As shown in Table 4, the ERA5 and GPT3 models are more suitable for stable and low-variability meteorological conditions, but perform poorly in the face of significant climate changes. Specifically, while ERA5 and GPT3 maintain good accuracy under stable climate conditions, they may fail to adapt adequately to rapid climate shifts or large seasonal variations, resulting in systematic biases. Nevertheless, ERA5 is still considered reliable reference standards, providing reasonably accurate ZTD estimates, particularly under stable conditions.

**Figure 6:**
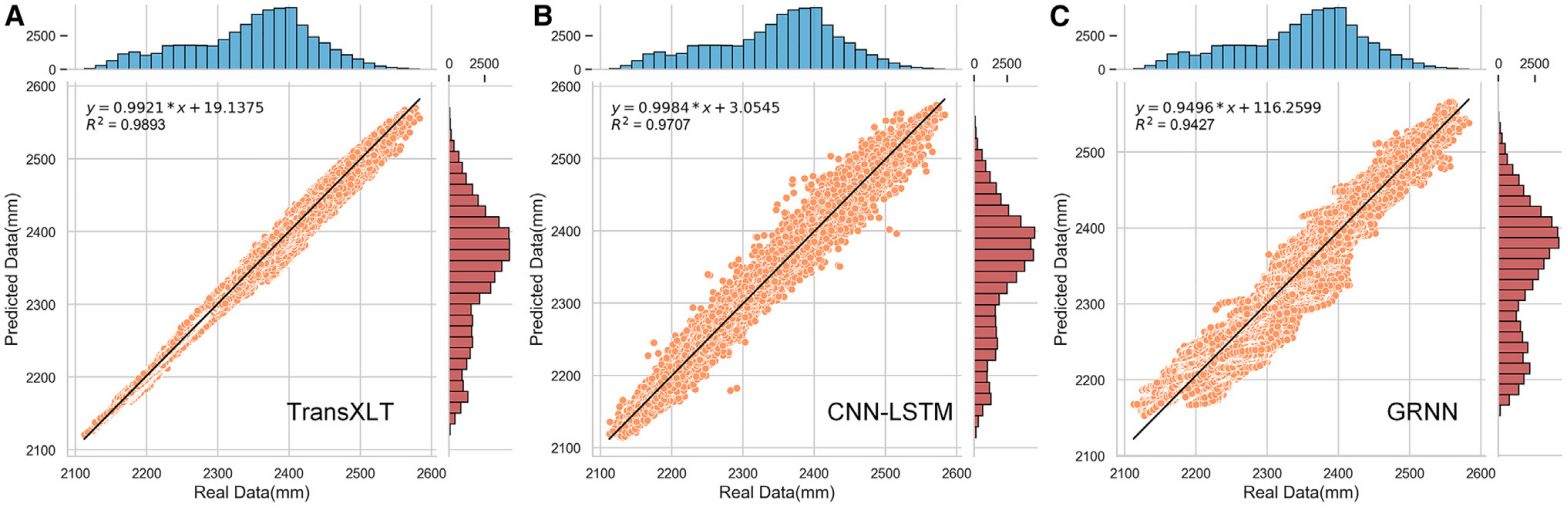
Scatterplot comparing predicted and true values (A) The scatterplot of prediction results from the TransXLT model. (B) The scatterplot of prediction results from the CNN-LSTM model. (C) The scatterplot of prediction results from the GRNN model.

We generated scatterplots for the three models, revealing that TransXLT achieves an *R*^2^ of 0.9893—surpassing CNN-LSTM (0.9707) and GRNN (0.9427)—which indicates the best fitting performance (Figure 6). CNN-LSTM shows slight error dispersion, while GRNN exhibits the largest error spread, particularly in extreme values. In summary, the TransXLT model demonstrates superior accuracy, linear consistency, and error distribution compared to CNN-LSTM and GRNN. It shows exceptional stability and precision, effectively capturing the complex fluctuation patterns of ZTD data.

### Model ablation experiment

To assess the performance advantages of the TransXLT framework compared to its individual components, a comprehensive ablation study was conducted using three configurations: (1) transformer-only: employing only the transformer module for temporal dependency modeling; (2) xLSTM-only: utilizing the extended LSTM architecture for sequential pattern learning; (3) TransXLT: combining both transformer and xLSTM via our proposed fusion mechanism. Evaluation metrics were based on the average values of bias, MAE, and RMSE across six test stations.

As shown in Table 5, the average values of bias, MAE, and RMSE for the TransXLT model are 0.50, 6.32, and 8.13 mm, respectively, significantly outperforming the individual components. Compared to the individual models, the combined TransXLT framework reduces the average RMSE by 31.5% and 38.6%. These results demonstrate that the TransXLT framework surpasses its individual components in model performance, with the improvement attributed to the complementary nature of transformer and xLSTM, which together capture long-term temporal dependencies and subtle inter-sequential variations.

**Table 5:** Ablation experiment results (mm)

Model	Mean Bias	Mean MAE	Mean RMSE	TransXLT accuracy improvement rate
Transformer-only	−1.64	8.63	11.87	69.5%/26.8%/31.5%
xLSTM-only	2.00	9.64	13.25	75.0%/34.4%/38.6%
TransXLT	0.50	6.32	8.13	–

### Influence of spatial factors on model accuracy

**Figure 7:**
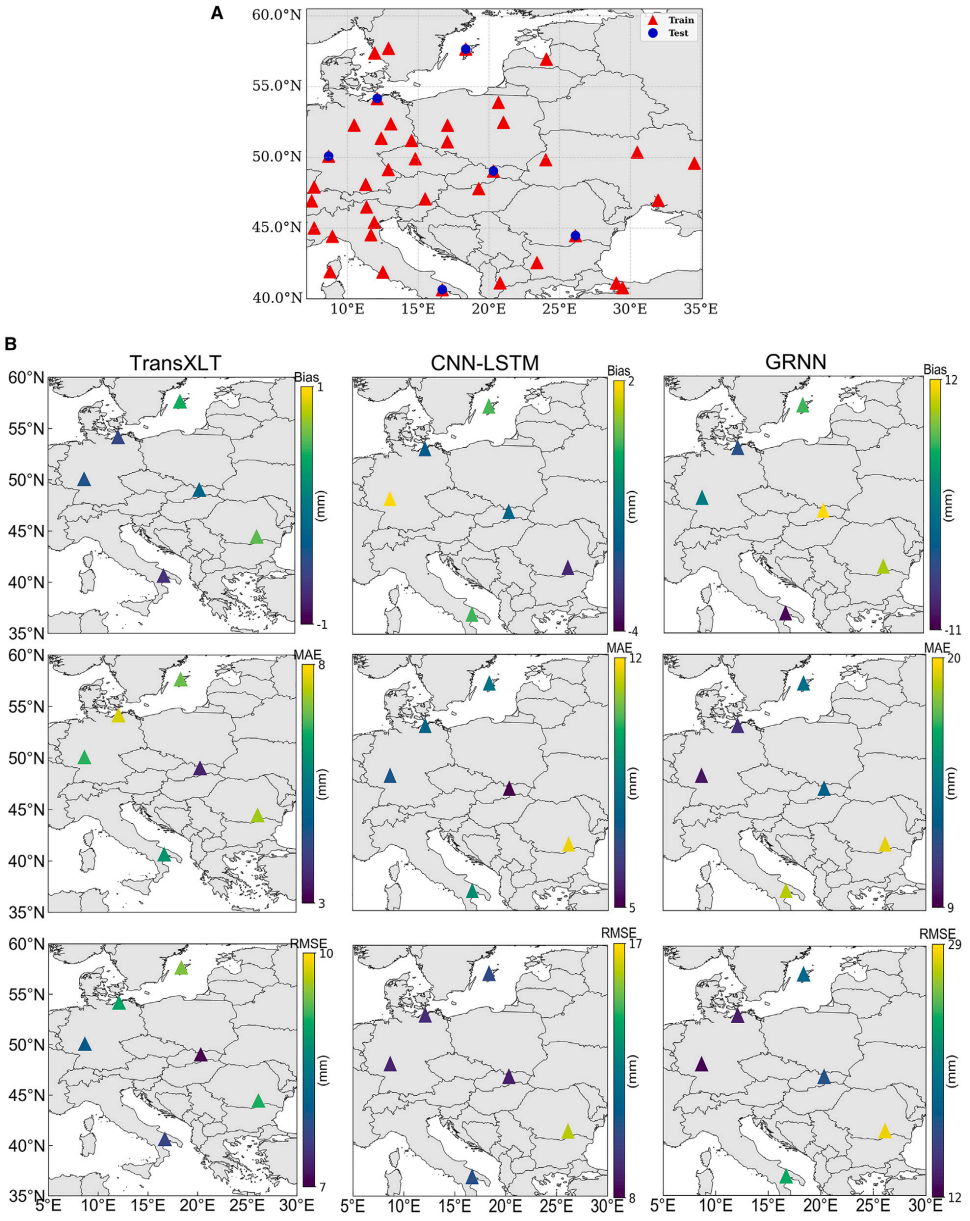
Impact of station locations on model accuracy (A) Shows the distribution of training and testing stations; (B) presents the errors obtained by three distinct models at each test station. From left to right, the results correspond to the TransXLT, CNN-LSTM, and GRNN models, and from top to bottom, they indicate Bias, MAE, and RMSE, respectively.

To evaluate the influence of station spatial locations on model accuracy, one station was selected as the test set based on its longitude, latitude, and spatial distribution relative to the training stations, while the remaining stations served as the training set. Each experiment used 38 stations for training and one for testing. The results are shown in Figure 7.

The error metrics across different stations are largely due to geographical variations. Stations near oceans or at higher latitudes experience larger meteorological changes and water vapor fluctuations, increasing model errors. Higher latitude, altitude, and proximity to coastal areas increase the likelihood of random temperature and humidity variations, causing larger prediction errors. Compared to coastal regions, inland stations have more stable seasonal changes in water vapor and temperature, leading to lower errors.

Notably, although the BUCU station is located in a relatively stable inland region, all three models present unexpectedly high RMSE values: 9.17 mm (TransXLT), 16.03 mm (CNN-LSTM), and 28.27 mm (GRNN). These results suggest that the density of training stations near the test station significantly influences model error. When fewer training stations are located near the test site, model errors tend to increase.

However, TransXLT mitigates these factors’ impact on model accuracy, showcasing superior adaptability and generalization capabilities. Test station results indicate that spatial factors influence RMSE by less than 3 mm.

### Model interpretability analysis

Shapley Additive Explanations (SHAP) is a game-theory-based method for explaining machine learning model predictions. By calculating each input feature’s contribution to prediction results, SHAP offers both global and local model explanations.^46^ We applied SHAP to quantify the contributions of input spatiotemporal features and prior constraint values to ZTD predictions. The calculation formula is as follows:

(Equation 1) $$\phi_{i}=\sum_{S\subseteq F\setminus \left\{i\right\}}\frac{\left|S\right|!\times \left(M-\left|S\right|-1\right)!}{M!}\times \left[f\left(S\cup \left\{i\right\}\right)-f\left(S\right)\right]$$

Where $\phi_{i}$ denotes the contribution of feature i to the model, F is the complete set of features, S⊆F∖{i} is a subset excluding, |S| is the subset size, and M is the total number of features. f(S) represents the model output using only the subset features, while f(S∪{i}) is the output after adding feature i to the subset.

**Figure 8:**
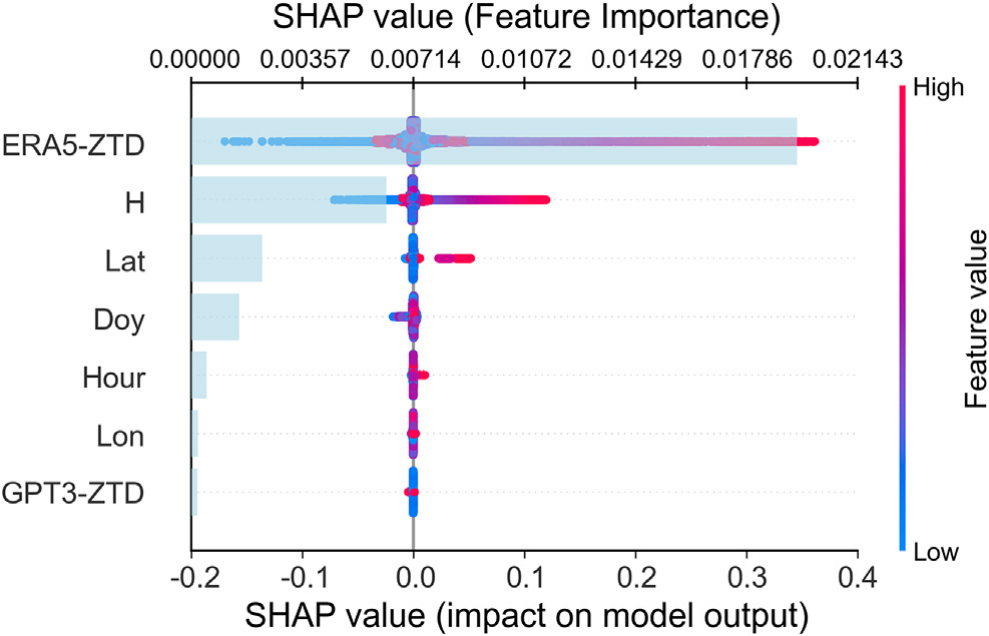
SHAP analysis of input features in the TransXLT model The light blue bars represent the importance of the input features, while the red and blue lines denote the impact of these features on the model’s output.

We present the contributions of various input features to the TransXLT model’s predictions (Figure 8). The vertical axis ranks feature importance in descending order, while the horizontal axis represents SHAP values, where positive values indicate a feature’s positive influence on predictions, and negative values indicate a diminishing effect. The point colors, ranging from blue (low) to red (high), represent the normalized input feature values, while the light blue bars denote feature importance.

From Figure 8, ERA5-ZTD and height (H) emerge as the two most influential features, contributing significantly and positively to the predictions, corroborating earlier conclusions. ERA5-ZTD, derived from meteorological reanalysis data, captures not only water vapor content but also key parameters like temperature, humidity, and atmospheric pressure. Since ZTD is highly sensitive to variations in water vapor and temperature, this comprehensive atmospheric representation is essential. As a result, ERA5-ZTD offers more accurate and detailed meteorological information, allowing the model to better capture spatiotemporal fluctuations in tropospheric delay.

Height influences ZTD due to the decrease in atmospheric density with increasing altitude. In low-altitude regions, the higher atmospheric density and water vapor content enhance the model’s ability to infer atmospheric variations. Height acts as an indirect indicator of climate, temperature, and humidity, particularly in varying geographical conditions.

Latitude contributes more than longitude, implying that it indirectly influences water vapor distribution and atmospheric density. This results in atmospheric delay variations at higher latitudes, highlighting latitude as a key feature for ZTD prediction. Time features have minimal impact, as ZTD values demonstrate pronounced annual and seasonal trends. The model’s use of a time window for input processing reduces the relevance of short-term temporal features. GPT3-ZTD shows minimal impact on the model’s predictions. In addition to its lower resolution and potential redundancy with ERA5-ZTD, GPT3 relies on global empirical formulas that may not be suitable for the specific climatic and geographic characteristics of the local study area. However, GPT3-ZTD is retained in the model as a complementary feature, as it provides additional atmospheric information that can enhance prediction robustness, especially in scenarios where ERA5-ZTD data may be limited or less reliable.

## Discussion

This study introduces the SASR model as a systematic solution for missing ZTD data in GNSS stations. In missing data reconstruction experiments, the SASR model exhibited significant advantages, achieving reconstruction accuracy far exceeding the traditional PCHIP method under high variability and complex climatic conditions, with an average error reduction of 24.5%. It is important to note that PCHIP relies on local piecewise cubic polynomials, making it computationally efficient for stable or low-frequency data but less capable of modeling rapid transitions. In contrast, SASR leverages a self-attention mechanism that can capture both local and long-range dependencies, enabling it to better handle high variability in ZTD time series. Consequently, while our results show a substantial advantage of SASR in high-fluctuation conditions, PCHIP can still serve as a straightforward baseline for situations where data patterns are more stable or smoothly varying. In future work, we plan to compare SASR with additional interpolation or data imputation methods to further validate its efficacy across a broader range of scenarios.

Meanwhile, we developed the TransXLT model by combining the Transformer architecture with extended LSTM, significantly enhancing its ability to extract spatiotemporal features of ZTD. The model leverages Doy, hour, latitude, longitude, height, GPT3-ZTD, and ERA5-ZTD as input features, enhancing its ability to model complex spatiotemporal variations. As illustrated in Figures 3 and 4 and Table 4, TransXLT outperforms traditional CNN-LSTM, GRNN, and the existing GPT3 and ERA5 models. Further analysis reveals that TransXLT mitigates the influence of geographical and climatic variations on ZTD accuracy, demonstrating greater consistency across different spatiotemporal conditions. SHAP value analysis identifies ERA5-ZTD and height as the most influential features, underscoring the importance of integrating multi-source meteorological and geographical data to enhance model performance. Additionally, TransXLT exhibits exceptional adaptability and robustness across varying latitudes, altitudes, and seasons, highlighting its broad applicability in complex meteorological and geographical environments.

Although the model demonstrates superior performance across most monitoring stations, some prediction errors may still occur under extreme climatic or complex geographical conditions. Furthermore, the model’s structural complexity may lead to prolonged training times over large-scale regions, thereby challenging its real-time applicability. This limitation is due to the reliance on data that often has inherent delays, such as the ERA5-ZTD, which may restrict the model’s performance in real-time settings. While the current version of the TransXLT model cannot operate in real-time scenarios, we see this as an area for future development. To facilitate the future expansion of the model to more complex terrains, we propose incorporating terrain features and spatial heterogeneity, thereby enhancing its adaptability and predictive accuracy in these environments. Specifically, integrating geographic information system (GIS) data, such as terrain elevation and slope factors, can enhance the model’s responsiveness to topographical changes. Additionally, adopting advanced training strategies like transfer learning or multi-task learning can leverage existing model knowledge from plain regions to support training in complex terrains, thereby enhancing the model’s generalization capability and robustness. We will also explore ways to adapt the model to real-time prediction systems by integrating near-real-time data, thereby enhancing the model’s real-time applicability and efficiency. Moreover, optimizing the model’s training efficiency and expanding its application potential in GNSS precise positioning, meteorological research, and other tropospheric delay-related fields are also among our future research objectives.

### Conclusion

This study introduces an innovative ZTD data reconstruction and prediction method, comprising the sparse attention-based reconstruction (SASR) model and TransXLT. The SASR model enhances missing data reconstruction accuracy through masked prediction and sparse self-attention mechanisms, showing strong adaptability in highly variable and seasonal conditions. Compared to traditional interpolation methods, SASR reduces MAE by 24.5%, ensuring high-quality input for predictive modeling. The TransXLT model efficiently predicts ZTD by integrating multi-source data (e.g., ERA5-ZTD and GPT3-ZTD) with spatiotemporal features (e.g., Doy, latitude, longitude, and height). Validated on data from stations outside the training set, the TransXLT model achieves average bias, MAE, and RMSE values of 0.50 mm, 6.32 mm, and 8.13 mm, respectively. It outperforms CNN-LSTM, GRNN, ERA5, and GPT3 models, reducing RMSE by 29.72%, 56.47%, 54.04%, and 76.54%, respectively. The model exhibits robust adaptability across varying latitudes, altitudes, and seasons, confirming its stability and effectiveness in complex environments.

In summary, this study introduces a novel technical framework for ZTD modeling, addressing data gaps and showcasing excellent performance in ZTD prediction tasks. The proposed approach demonstrates significant potential for application, particularly in complex geographical and meteorological conditions. Future research will aim to optimize the model’s efficiency and investigate its broader applicability and adaptability in practical scenarios.

### Limitations of the study

The predictive model proposed in this study is based on post-hoc data, as our input features are derived from retrospective products. Consequently, the model is not immediately applicable for real-time tropospheric delay forecasting; achieving this would require substituting these features with appropriately matched real-time data sources—a goal we intend to pursue in future work. Additionally, our study did not account for the influence of regional terrain factors on model accuracy. Future research will focus on developing a comprehensive global ZTD prediction model that incorporates terrain variability to further enhance predictive performance.

## Resource availability

### Lead contact

Further information and requests for resources should be directed to Xuexiang Yu (yuxx_aust@163.com).

### Materials availability

This study did not generate new unique materials.

### Data and code availability

• This paper analyzes existing, publicly available data. The specific repository links and accession numbers are listed in the key resources table. The raw IGS-ZTD data used in this study are available in the supplemental Data S1.
• Due to ongoing research and contractual agreements, the full code cannot be publicly released at this stage. We anticipate making the code available after the completion of the project (i.e., June 2026). If further information is required to replicate the core methodology, please contact the lead contact. The original code for the baseline models used in this study is publicly available, and the accession numbers are listed in the key resources table.
• Any additional information required to reanalyze the data reported in this paper is available from the lead contact upon request.

## Acknowledgments

This work was supported by the 10.13039/501100001809National Natural Science Foundation of China (no. 42304050), the Key R&D Program of Anhui Province (no. 202104a07020014), the Major S&T Projects of Anhui Province (no. 202103a05020026), and the Coal Industry Engineering Research Center for Mining Area Environmental and Disaster Monitoring (no. KSXTJC202207). Additional support was provided by the Scientific Research Foundation for High-level Talents of Anhui University of Science and Technology (no. 2022yjrc66), the Key Laboratory of Space–Earth Cooperative Monitoring and Early Warning of Mining-Induced Disasters (no. KLAHEI202202), and the Engineering Research Center for Collaborative Monitoring of Mining Environment and Disasters in the Coal Industry (no. KSXTJC202303).

## Author contributions

Conceptualization, S.X. and X. Yu; methodology, S.X., X. Yang, and J.Y.; validation, S.X., X. Yu, and M.Z.; investigation, S.X. and Y.H.; data curation, S.X., M.W., and Z.G.; writing-original draft, S.X.; writing-review and editing, all authors.; visualization, S.X. and X. Yang; supervision, X.Y. and J.Y.; funding acquisition, X.Y., J.Y., X. Yang, and Z.G.

## Declaration of interests

The authors declare no competing interests.

## STAR★Methods

### Key resources table

**Table:** 

REAGENT or RESOURCE	SOURCE	IDENTIFIER
**Deposited data**		

GNSS data with a 5-min temporal resolution	International GNSS Service	Data S1
GPT3 data with a spatiotemporal resolution of 1 h/1°	VMF Data Server	https://vmf.geo.tuwien.ac.at/codes/
ERA5 data with a spatial resolution of 1 h/0.25°	European Center for Medium-Range Weather Forecasts	https://cds.climate.copernicus.eu/datasets/reanalysis-era5-pressure-levels

**Software and algorithms**		

Python (version 3.9)	Python Software Foundation	https://www.python.org/
PyTorch (version 1.13.1)	PyTorch Foundation	https://pytorch.org/
MATLAB (version R2023b)	MathWorks	https://www.mathworks.com/products/matlab.html
Transformer	Zhang et al.^28^	https://github.com/hyunwoongko/transformer.git
xLSTM	Beck et al.^33^	https://github.com/kyegomez/xLSTM.git

### Method details

#### Data sources and ZTD extraction

This study focuses on the European Plain, a region spanning 7°E−35°E and 40°*N*–58°N (Figure S1). The region features flat terrain and low topographical complexity, which minimizes terrain interference in ZTD prediction, thereby allowing more accurate assessment and validation of model performance. Furthermore, the European Plain exhibits typical meteorological conditions, encompassing various climate types that provide rich and diverse data for model training and validation. Additionally, the area boasts a well-established observation system, offering high-quality ZTD data from numerous monitoring stations, thereby providing a solid foundation for model training and validation. These factors make it an ideal region for tropospheric modeling research. The study utilizes GNSS tropospheric delay data from 39 active IGS stations in the region (January 1–December 31, 2022), GPT3 grid data, and ECMWF ERA5 reanalysis data. The dataset details in the key resources table.

#### GNSS tropospheric delay products

Since 1997, the International GNSS Service (IGS) has been providing post-processed tropospheric correction products. By 2020, its global monitoring network included over 400 GNSS stations delivering daily tropospheric data, such as 5-min ZTD values, northward and eastward gradients, and station coordinates.^47^ For this study, ZTD products from 39 European GNSS stations were used as ground truth to train and evaluate the model. To match the temporal resolution of GPT3 and ERA5 data, we downsampled the ZTD products through an hourly averaging method. The original 5-min interval data were aggregated into hourly IGS-ZTD values, thereby creating a standardized dataset with a unified temporal scale for model input.

#### Global Pressure and Temperature 3 model

Landskron and Bohm introduced the GPT3 model in 2018, the latest and most accurate version of the GPT series for empirical tropospheric delay modeling.^14^^,^^48^^,^^49^ The model provides a comprehensive range of tropospheric parameters, including weighted mean temperature ($T_{m}$), temperature (T), atmospheric pressure (P), water vapor pressure (e), temperature lapse rate (dT), water vapor lapse rate (λ), and hydrostatic and wet mapping functions derived from the VMF3 model. GPT3 enhances GPT2w by retaining its meteorological parameters while adding north and east gradients. The data are available in global geographic grids at resolutions of 1 ° × 1 ° and 5 ° × 5 °. Users can retrieve meteorological parameters for a specific location and time by inputting the station’s geo-graphic coordinates and time information. The calculation formula is shown below.^14^

(Equation 2) $$\begin{array}{l} r\left(t\right)=A_{0}+A_{1}\times \cos\left(\frac{doy}{365.25}\cdot 2\pi \right)+B_{1}\times \sin\left(\frac{doy}{365.25}\cdot 2\pi \right)+\ldots \\ A_{2}\times \cos\left(\frac{doy}{365.25}\cdot 4\pi \right)+B_{2}\times \sin\left(\frac{doy}{365.25}\cdot 4\pi \right) \end{array}$$

r(t) represents the meteorological parameters from the GPT3 model, doy is the day of the year, $A_{0}$ is the mean value, and $\left(A_{1},B_{1}\right)$, $\left(A_{2},B_{2}\right)$ are the annual and semiannual amplitudes, respectively. Meteorological parameters from the four closest grid points to the station are interpolated using bilinear interpolation. Using the station’s meteorological parameters, the Saastamoinen model^5^ and the Askne-Nordius model^50^ calculate the ZTD. The formulas are as follows:

(Equation 3) $$ZHD=\frac{0.0022768\times P}{1-0.00266\times \cos\left(2\phi \right)-0.00028\times H}$$

(Equation 4) $$ZWD=10^{-6}\left(k_{2}^{\prime }+\frac{k_{3}}{T_{m}}\right)\frac{R_{d}}{\left(\lambda +1\right)g_{m}}e$$

(Equation 5) ZTD=ZHD+ZWD

Here, ϕ and H denote the station’s latitude and ellipsoidal height, respectively. $k_{2}^{\prime }$ (16.52 K/hPa) and $k_{3}$ (3.776 × 10^5^ K^2^/hPa) are atmospheric refraction constants, and $R_{d}$ (287.0464 J K^−1^ kg^−11^) is the specific gas constant for dry air. The mean gravitational acceleration $g_{m}$ (9.80655 m/s^2^) is considered. The definitions of other parameters are provided above. In this study, the ZTD derived from GPT3 1 ° × 1 ° grid data (GPT3_1) is termed GPT3-ZTD. Compared to GPT3 5 ° × 5 ° grid data (GPT3_5), GPT3_1 offers marked improvements in stability and accuracy.^51^

#### ECMWF fifth generation reanalysis data

ERA5, offered by the European Center for Medium-Range Weather Forecasts (ECMWF), represents the fifth generation of atmospheric reanalysis products. Featuring high temporal and spatial resolution, comprehensive meteorological variables, and enhanced process representation, ERA5 serves as a vital data source for atmospheric research and tropospheric delay modeling.^9^ The ERA series has continuously evolved since the release of the first-generation reanalysis product ERA-15 in 1979. Updated in January 2019, ERA5 offers a temporal resolution of 1 h and a horizontal resolution of 0.25 ° × 0.25 °.

This study utilized ERA5’s hourly pressure-level data product. Station-specific meteorological parameters were derived using bilinear interpolation horizontally and linear interpolation vertically. The ZTD at each station was computed by combining the model method and the refractivity integral method. The specific steps are as follows.

(1) Extract meteorological parameters from the ERA5 product, which spans 37 standard pressure levels. Since GNSS stations use ellipsoidal heights as references, the Earth Gravitational Model 2008 (EGM2008) is applied to convert ellipsoidal heights into geopotential heights, aligning them with ERA5 meteorological parameters.^52^
(2) Interpolate meteorological parameters to GNSS station locations. Horizontally, bi-linear interpolation is conducted using the station’s latitude and longitude with the four nearest grid points. Vertically, if the station is located above the lowest pressure level, temperature and specific humidity are interpolated linearly, while pressure is calculated using the exponential model:^53^

(Equation 6) $$\left\{ \begin{array}{l} P\left(h\right)=0.01P_{0}\;\exp\left(\frac{-g\cdot \Delta h}{R_{d}\cdot T_{v}}\right)\\ T_{v}=T_{0}\left(1+0.6077q_{0}\right) \end{array}\right.$$

Where, P(h) represents pressure at the station (hPa); $P_{0}$ , $T_{0}$ , $q_{0}$ denote pressure, temperature (K), and specific humidity at the nearest grid points, respectively; g refers to gravitational acceleration; Δh indicates the height difference between the station and the grid point; $R_{d}$ denotes the specific gas constant for dry air (8.3143 J/K·mol); $T_{v}$ represents virtual temperature.

If the station is situated below the lowest ERA5 pressure layer (i.e., below sea level or the minimum height of the pressure layers), temperature and pressure are extrapolated,^54^ while specific humidity remains unchanged. The formula is given as:

(Equation 7) $$\left\{ \begin{array}{l} T\left(h\right)=T_{0}+\Gamma \cdot \left(h-h_{0}\right)\\ P\left(h\right)=P_{0}\times \exp\left(-\frac{g\cdot \left(h_{0}-h\right)}{R_{d}\cdot T_{v}}\right)\\ Q\left(h\right)=Q_{0} \end{array}\right.$$

Where, T(h) , Q(h) represent the temperature and specific humidity at the station; $T_{0}$, $h_{0}$, $P_{0}$, $Q_{0}$ denote the temperature, height, pressure, and specific humidity at the lowest pressure layer, respectively; h indicates the station height; Γ (−6.5 K/km) refers to the standard atmospheric temperature lapse rate.

(3) Using the interpolated specific humidity $Q_{s}$ and pressure $P_{s}$, the water vapor pressure e and refractivity N are calculated layer by layer as follows:

(Equation 8) $$\left\{ \begin{array}{l} e=\frac{Q_{s}\times P_{s}}{0.62198+0.37802q}\\ N=K_{1}\cdot \frac{P_{s}-e}{T_{s}}+K_{2}\cdot \frac{e}{T_{s}}+K_{3}\cdot \frac{e}{T_{s}^{2}} \end{array}\right.$$

Where, $K_{1}$, $K_{2}$, $K_{3}$ are atmospheric refraction coefficients with values of 77.604 K/hPa, 64.79 K/hPa, and 377600 K^2^/hPa, respectively.

(4) Refractivity is integrated along the zenith direction up to the top of the isobaric surface. Since the wet delay effect above the tropopause is negligible, the ZTD in this region is calculated using the Saastamoinen dry delay model, as shown below:

(Equation 9) $$\left\{ \begin{array}{l} ZTD_{level}=10^{-6}\int_{H}^{H_{top}}NdH\\ ZTD_{Saasta}=0.002277\times \frac{P+\left(\frac{1255}{T}+0.05\right)e}{1-0.00266\times \cos\left(2\phi \right)-0.00028H_{top}} \end{array}\right.$$

Where H denotes the geopotential height of the station, $H_{top}$ represents the tropopause height, and P, T, e stand for the pressure, temperature, and water vapor pressure at the corresponding location.

(5) Finally, the two ZTD components are summed to obtain the station’s ZTD, known as ERA5-ZTD.

(Equation 10) $$ZTD=ZTD_{level}+ZTD_{Saasta}$$

#### Reconstruction of missing data based on sparse self-attention

Due to network failures and other factors, ZTD products from International GNSS Service (IGS) may have gaps lasting from hours to days. When GNSS stations have missing observations, ZTD for those periods cannot be retrieved. Data quality and completeness are crucial for accurate time series modeling.^55^ We selected 33 GNSS station data for model training in 2022 and performed data completeness statistics (Figure S2). All stations show varying levels of data gaps, with the WTZA station having a completeness rate of just 72.3%. Effectively handling missing data is essential to ensure modeling accuracy.

This study introduces a Sparse Attention-based Time Series Reconstruction Model (SASR) utilizing the self-attention mechanism.^56^ The model simultaneously handles two tasks: masked prediction and data reconstruction.

In the first task, the model’s ability to handle partial data loss is evaluated by randomly masking observed data and predicting the missing values, simulating real-world data loss. Performance is assessed using the mean absolute error (MAE) between predicted and actual masked values.

The second task evaluates the model’s ability to reconstruct observed data while maintaining consistency. The model must interpolate missing values without compromising the integrity of the complete dataset. Accuracy is measured by minimizing the MAE between reconstructed and original values, highlighting the model’s capacity to predict missing data without introducing bias.

The SASR model utilizes a standard Transformer encoder architecture, consisting of two sparse self-attention modules and a weighted combination module (Figure S3). To enhance computational efficiency for long sequences and focus on key temporal dependencies, the model incorporates a Top-k sparsification strategy within the self-attention mechanism. In this strategy, only the top k key-value pairs with the highest attention weights are retained, reducing time complexity from *O*(*L*^2^) to *O*(*Lk*). The value of k is dynamically adjusted during training based on sequence length to capture the most important dependencies without excessive computation. This strategy minimizes redundant computations while preserving the most relevant temporal dependencies across extended time series.

The input time series is first projected into an internal model dimension *d*_model_ through a fully connected layer, with positional encoding incorporated to embed temporal location information. The encoded data are then fed sequentially through two sparse attention blocks. Each block contains a sparse attention sublayer that captures essential temporal dependencies and a feedforward sublayer that extracts higher-level features. To ensure model stability and prevent gradient issues, residual connections and layer normalization are applied after each sublayer.

These two attention blocks generate intermediate outputs, denoted as Representation 1 and Representation 2. Representation 1 corresponds to the preliminary interpolated output from the first block, while Representation 2 is a refined output that enhances temporal feature extraction through further processing in the second block.

In the final stage, the weighted combination module dynamically fuses Representation 1 and Representation 2 using learnable attention weights. These weights are modulated through a linear transformation followed by the Sigmoid function to balance the contributions of each output. During training, the loss function combines the errors from the data masking and data reconstruction tasks using an adjustable weighting coefficient λ, which controls the error contribution from each task. In the experiments, we set λ to 1.The combined representation, Representation 3, is passed through a final layer to produce the interpolated time series. This output accurately reconstructs both missing and observed data, thereby improving the model’s performance in time series reconstruction tasks.

#### Building the Transformer-xLSTM prediction model

In constructing the ZTD prediction model, spatiotemporal features from training stations serve as inputs, with predicted ZTD as the output. Input features include Day of Year (Doy), Hour, Longitude (Lon), Latitude (Lat), Ellipsoidal Height (H), GPT3-ZTD, and ERA5-ZTD (detailed calculation methods for these can be found in the method details section). ERA5 provides high-resolution global meteorological data, effectively capturing atmospheric spatiotemporal variations, while GPT3 offers empirical estimates based on atmospheric pressure and temperature, assisting in predicting and repairing missing data. By combining these data sources into a prior ZTD constraint, the model can capture ZTD variations more accurately. A sliding window mechanism further segments data at each time step into time series inputs, improving the model’s ability to capture temporal dependencies and sequential patterns.

To meet ZTD modeling requirements, we enhanced the traditional Transformer architecture, resulting in the TransXLT model (Figure S4). These innovations aim to improve model performance and generalization, balancing accuracy and computational efficiency.

Improvement 1: An xLSTM was introduced, based on the traditional LSTM structure. xLSTM consists of two components: sLSTM and mLSTM. sLSTM employs a scalar memory up-date mechanism for fine-grained control of memory cells, enhancing model stability with long sequence data. mLSTM boosts memory capacity and parallel processing through covariance matrix updates and normalization.

Improvement 2: The xLSTM and Transformer are integrated in the following way: the xLSTM module first extracts initial temporal features from the input sequence, and then the Transformer’s self-attention layers capture these representations to compute global dependencies.

Improvement 3: The traditional decoder layer was replaced by the Transformer encoder layer for feature extraction. In addition, two 1D convolutional pooling layers (Conv1D Pooling) and a dense layer were introduced to enhance feature representation, reduce redundancy, and improve computational efficiency. Unlike conventional fixed pooling operations (e.g., max pooling), Conv1D Pooling employs learnable convolution kernels that can adaptively aggregate local features, thereby preserving essential information and capturing local dependencies more effectively.

Improvement 4: Dropout and layer normalization were applied in the encoder layer to prevent overfitting, while a 10-fold cross-validation strategy provided insights into potential overfitting and guided adjustments of dropout rates and normalization parameters. Finally, the ReLU activation function and a linear layer mapped the features to the output space, generating predictions.

### Quantification and statistical analysis

The model’s performance was assessed using four metrics: Bias, mean absolute error (MAE), root-mean-square error (RMSE), and the coefficient of determination (R^2^). The corresponding formulas are as follows.

(Equation 11) $$\text{Bias}=\sum_{i=1}^{n}\left({\text{ZTDpre}}_{i}-{\text{ZTDrea}}_{i}\right)/n$$

(Equation 12) $$\text{MAE}=\sum_{i=1}^{n}|{\text{ZTDpre}}_{i}-{\text{ZTDrea}}_{i}|/n$$

(Equation 13) $$\text{RMSE}=\sqrt{\sum_{i=1}^{n}{\left({\text{ZTDpre}}_{i}-{\text{ZTDrea}}_{i}\right)}^{2}/n}$$

(Equation 14) $$R^{2}=1-\frac{\sum_{i=1}^{n}{\left({\text{ZTDpre}}_{i}-{\text{ZTDrea}}_{i}\right)}^{2}}{\sum_{i=1}^{n}{\left({\text{ZTDrea}}_{i}-\bar{\text{ZTDrea}}\right)}^{2}}$$

In this formula, ZTDpre and ZTDrea denote the predicted ZTD and IGS-ZTD, respectively, and n represents the total number of samples.

## Supplemental Information

Document S1. Figures S1–S4

Data S1. Raw IGS-ZTD data used in the study, related to STAR Methods

## References

[1] Rózsa S., Ambrus B., Juni I., Ober P.B., Mile M. (2020). An advanced residual error model for tropospheric delay estimation. GPS Solut..

[2] Nzelibe I.U., Tata H., Idowu T.O. (2023). Assessment of GNSS zenith tropospheric delay responses to atmospheric variables derived from ERA5 data over Nigeria. Satell. Navig..

[3] Yang F., Guo J., Meng X., Li J., Zou J., Xu Y. (2021). Establishment and assessment of a zenith wet delay (ZWD) augmentation model. GPS Solut..

[4] Li J., Yao Y., Liu L., Zhang B., Huang L., Cao L. (2023). A predicting ZWD model based on multi-source data and generalized regression neural network. Acta Geod. Cartogr. Sinica.

[5] Saastamoinen J. (2013). Atmospheric correction for the troposphere and stratosphere in radio ranging satellites. The Use of Artificial Satellites for Geodesy.

[6] Hopfield H.S. (1969). Two-quartic tropospheric refractivity profile for correcting satellite data. J. Geophys. Res..

[7] Hopfield H.S. (1971). Tropospheric Effect on Electromagnetically Measured Range: Prediction from Surface Weather Data. Radio Sci..

[8] Zhang L., Zhang Q., Yao Y., Yu S., Wang W., Yang A. (2023). Refining empirical tropospheric model with meteorological stations for large height difference RTK positioning. GPS Solut..

[9] Huang L., Lu D., Chen F., Zhang H., Zhu G., Liu L. (2024). A Deep Learning-Based Approach for Directly Retrieving GNSS Precipitable Water Vapor and Its Application in Typhoon Monitoring. IEEE T Geosci Remote.

[10] Xu Y., Yang Z., Zhou H., Zhang F. (2024). An initial investigation of the non-isotropic feature of GNSS tropospheric delay. Satell. Navig..

[11] Leandro R.F., Langley R.B., Santos M.C. (2008). UNB3m_pack: a neutral atmosphere delay package for radiometric space techniques. GPS Solut..

[12] Guo Q., Wu X. (2019). A global assessment of ray-traced and blind tropospheric models in the retrieval of tropospheric parameters from ground-based GPS observations. J Atmos Sol-Terr Phy.

[13] Huang L., Zhu G., Peng H., Liu L., Ren C., Jiang W. (2022). An improved global grid model for calibrating zenith tropospheric delay for GNSS applications. GPS Solut..

[14] Landskron D., Böhm J. (2018). VMF3/GPT3: refined discrete and empirical troposphere mapping functions. J. Geod..

[15] Ding J., Chen J., Wang J., Zhang Y. (2024). A novel method for tropospheric delay mapping function vertical modeling. J. Geod..

[16] Yao Y., He C., Zhang B., Xu C. (2013). A new global zenith tropospheric delay model GZTD. Chinese J Geophys-Ch.

[17] Yao Y., Hu Y., Yu C., Zhang B., Guo J. (2016). An improved global zenith tropospheric delay model GZTD2 considering diurnal variations. Nonlinear Proc Geoph.

[18] Sun Z., Zhang B., Yao Y. (2019). A global model for estimating tropospheric delay and weighted mean temperature developed with atmospheric reanalysis data from 1979 to 2017. Remote Sens..

[19] Yang H., Hu W., Yu L., Nie X., Li H. (2020). GHop: a new regional tropospheric zenith delay model. Geomatics Inf. Sci. Wuhan Univ..

[20] W L., Y Y., J O., Y H. (2018). IGGtrop_SH and IGGtrop_rH: Two Improved Empirical Tropospheric Delay Models Based on Vertical Reduction Functions. IEEE T Geosci Remote.

[21] Mateus P., Catalão J., Mendes V.B., Nico G. (2020). An ERA5-Based Hourly Global Pressure and Temperature (HGPT) Model. Remote Sens..

[22] Huang L., Lan S., Zhu G., Chen F., Li J., Liu L. (2023). A global grid model for the estimation of zenith tropospheric delay considering the variations at different altitudes. Geosci. Model Dev. (GMD).

[23] Li L., Xu Y., Yan L., Wang S., Liu G., Liu F. (2020). A Regional NWP Tropospheric Delay Inversion Method Based on a General Regression Neural Network Model. Sensors-Basel.

[24] Crocetti L., Schartner M., Zus F., Zhang W., Moeller G., Navarro V., See L., Schindler K., Soja B. (2024). Global, spatially explicit modelling of zenith wet delay with XGBoost. J. Geod..

[25] Mohammed J. (2021). Artificial neural network for predicting global sub-daily tropospheric wet delay. J Atmos Sol-Terr Phy.

[26] Wu Y., Huang L., Feng W., Tian S. (2024). A Hybrid Deep Learning Algorithm for Tropospheric Zenith Wet Delay Modeling with the Spatiotemporal Variation Considered. Atmosphere-Basel.

[27] Lu C., Zheng Y., Wu Z., Zhang Y., Wang Q., Wang Z., Liu Y., Zhong Y. (2023). TropNet: a deep spatiotemporal neural network for tropospheric delay modeling and forecasting. J. Geod..

[28] Zhang H., Yao Y., Xu C., Xu W., Shi J. (2022). Transformer-Based Global Zenith Tropospheric Delay Forecasting Model. Remote Sens..

[29] Hu F., Sha Z., Wei P., Xia P., Ye S., Zhu Y., Luo J. (2024). Deep learning for GNSS zenith tropospheric delay forecasting based on the informer model using 11-year ERA5 reanalysis data. GPS Solut..

[30] Wu H., Xu J., Wang J., Long M. (2021). Autoformer: Decomposition transformers with auto-correlation for long-term series forecasting. Adv. Neural Inf. Process. Syst..

[31] Zhou T., Ma Z., Wen Q., Wang X., Sun L., Jin R. (2022).

[32] Liu Y., Hu T., Zhang H., Wu H., Wang S., Ma L., Long M. (2023). itransformer: Inverted transformers are effective for time series forecasting. arXiv.

[33] Beck M., Pöppel K., Spanring M., Auer A., Prudnikova O., Kopp M., Klambauer G., Brandstetter J., Hochreiter S. (2024). xLSTM: Extended Long Short-Term Memory. arXiv.

[34] Yuan P., Blewitt G., Kreemer C., Hammond W.C., Argus D., Yin X., Van Malderen R., Mayer M., Jiang W., Awange J., Kutterer H. (2023). An enhanced integrated water vapour dataset from more than 10 000 global ground-based GPS stations in 2020. Earth Syst. Sci. Data.

[35] Shi J., Li X., Li L., Ouyang C., Xu C. (2023). An Efficient Deep Learning-Based Troposphere ZTD Dataset Generation Method for Massive GNSS CORS Stations. IEEE T Geosci Remote.

[36] Konakoglu B., Kutlu Onay F., Aydemir S.B. (2023). Tropospheric zenith wet delay prediction with a new hybrid ANN – Gorilla troops optimizer algorithm. Adv. Space Res..

[37] García-Laencina P.J., Sancho-Gómez J.-L., Figueiras-Vidal A.R., Verleysen M. (2009). K nearest neighbours with mutual information for simultaneous classification and missing data imputation. Neurocomputing.

[38] Selbesoglu M.O. (2019). Spatial Interpolation of GNSS Troposphere Wet Delay by a Newly Designed Artificial Neural Network Model. Appl Sci-Basel.

[39] Zus F., Douša J., Kačmařík M., Václavovic P., Balidakis K., Dick G., Wickert J. (2019). Improving GNSS Zenith Wet Delay Interpolation by Utilizing Tropospheric Gradients: Experiments with a Dense Station Network in Central Europe in the Warm Season. Remote Sens..

[40] Huang L., Bi H., Zhang H., Wang S., Liao F., Liu L., Jiang W. (2024). An optimized BP neural network for modeling zenith tropospheric delay in the Chinese mainland using coupled particle swarm and genetic algorithm. Geo Spatial Inf. Sci..

[41] Li S., Xu T., Xu Y., Jiang N., Bastos L. (2022). Forecasting gnss zenith troposphere delay by improving gpt3 model with machine learning in antarctica. Atmosphere-Basel.

[42] Li Q.Z., Böhm J., Yuan L.G., Weber R. (2024). Global zenith wet delay modeling with surface meteorological data and machine learning. GPS Solut..

[43] Zhang B., Yao Y. (2021). Precipitable water vapor fusion based on a generalized regression neural network. J. Geod..

[44] He L., Yao Y., Xu C., Zhang H., Tang F., Ji C., Liu Z., Wu W. (2024). A New Global ZTD Forecast Model Based on Improved LSTM Neural Network. IEEE J-Stars.

[45] Li W., Liu C., Xu Y., Niu C., Li R., Li M., Hu C., Tian L. (2024). An interpretable hybrid deep learning model for flood forecasting based on Transformer and LSTM. J Hydrol-Reg Stud.

[46] Baptista M.L., Goebel K., Henriques E.M.P. (2022). Relation between prognostics predictor evaluation metrics and local interpretability SHAP values. Artif. Intell..

[47] Li S., Xu T., Jiang N., Yang H., Wang S., Zhang Z. (2021). Regional Zenith Tropospheric Delay Modeling Based on Least Squares Support Vector Machine Using GNSS and ERA5 Data. Remote Sens..

[48] Lagler K., Schindelegger M., Böhm J., Krásná H., Nilsson T. (2013). GPT2: Empirical slant delay model for radio space geodetic techniques. Geophys. Res. Lett..

[49] Böhm J., Möller G., Schindelegger M., Pain G., Weber R. (2015). Development of an improved empirical model for slant delays in the troposphere (GPT2w). GPS Solut..

[50] Askne J., Nordius H. (1987). Estimation of tropospheric delay for microwaves from surface weather data. Radio Sci..

[51] Zhao Q., Su J., Xu C., Yao Y., Zhang X., Wu J. (2023). High-precision ZTD model of altitude-related correction. IEEE J-Stars.

[52] Zhu G., Huang L., Yang Y., Li J., Zhou L., Liu L. (2022). Refining the ERA5-based global model for vertical adjustment of zenith tropospheric delay. Satell. Navig..

[53] Yao Y., Xu C., Shi J., Cao N., Zhang B., Yang J. (2015). ITG: A new global GNSS tropospheric correction model. Sci. Rep..

[54] Böhm J., Heinkelmann R., Schuh H. (2007). Short note: a global model of pressure and temperature for geodetic applications. J. Geod..

[55] Thakur S., Choudhary J., Singh D.P. (2021).

[56] Du W., Côté D., Liu Y. (2023). Saits: Self-attention-based imputation for time series. Expert Syst. Appl..

