# Supplementary material for: TransXLT: A novel ZTD prediction method with SASR-based data reconstruction
Source: iScience. 2025 Mar 31;28(5):112328. doi: 10.1016/j.isci.2025.112328 (PMC12019023; doi:10.1016/j.isci.2025.112328)
Supplement: Document S1. Figures S1–S4 [file mmc1.pdf]

iScience, Volume *28*

Supplemental information

TransXLT: A novel ZTD prediction method with SASR-based data reconstruction

Shicheng Xie, Xuexiang Yu, Jiajia Yuan, Xu Yang, Mingfei Zhu, Yuchen Han, Min Wei, and Zhongchen Guo

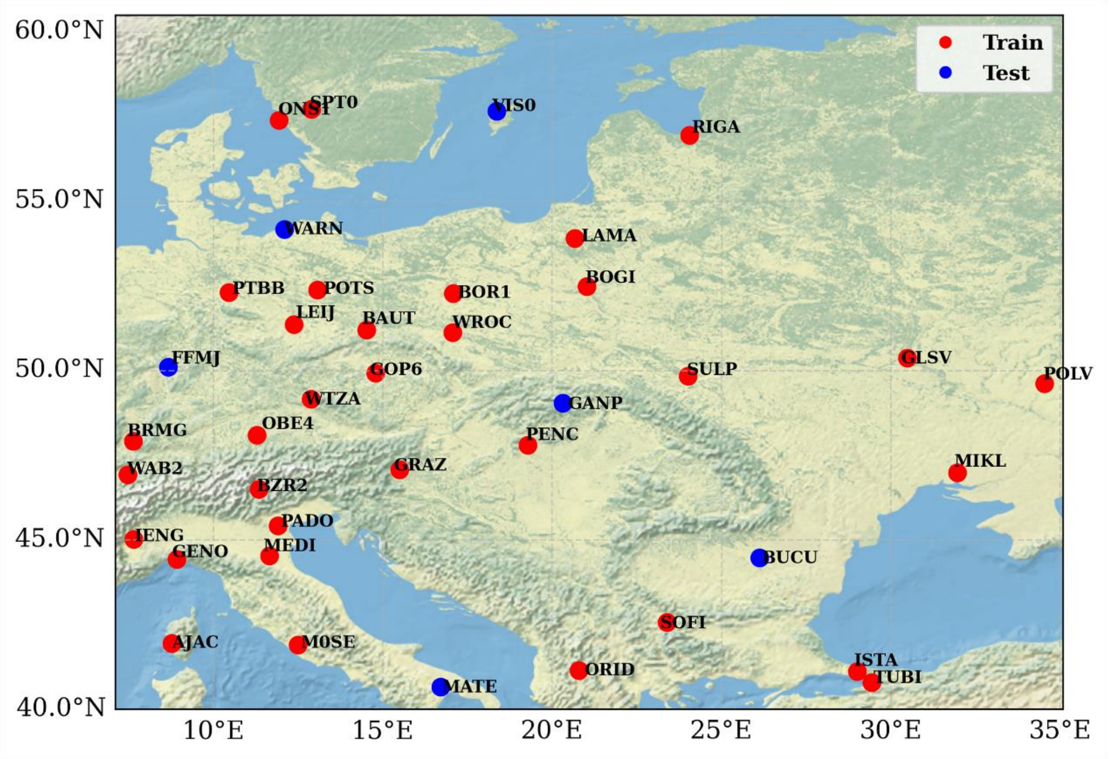

Figure S1. The GNSS station distribution used in this study, related to STAR Methods. Red circles denote training stations and blue circles denote testing stations.

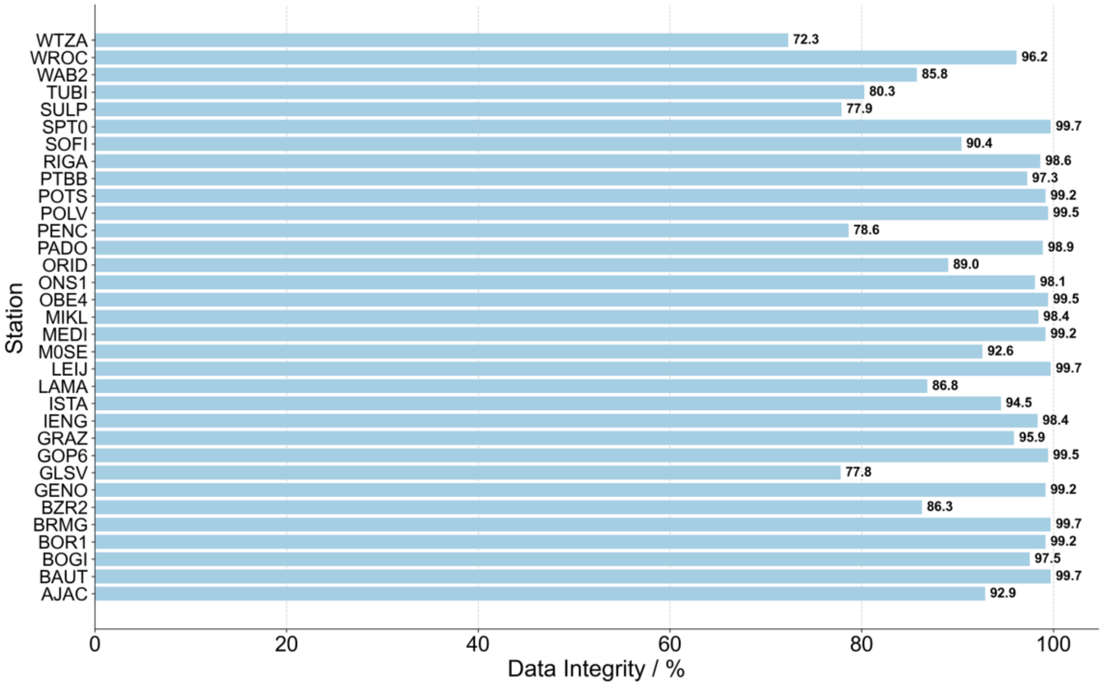

Figure S2. ZTD data integrity rate of 33 measurement stations, related to STAR Methods. Data integrity (%) is calculated as the proportion of valid ZTD observations at each station over the study period.

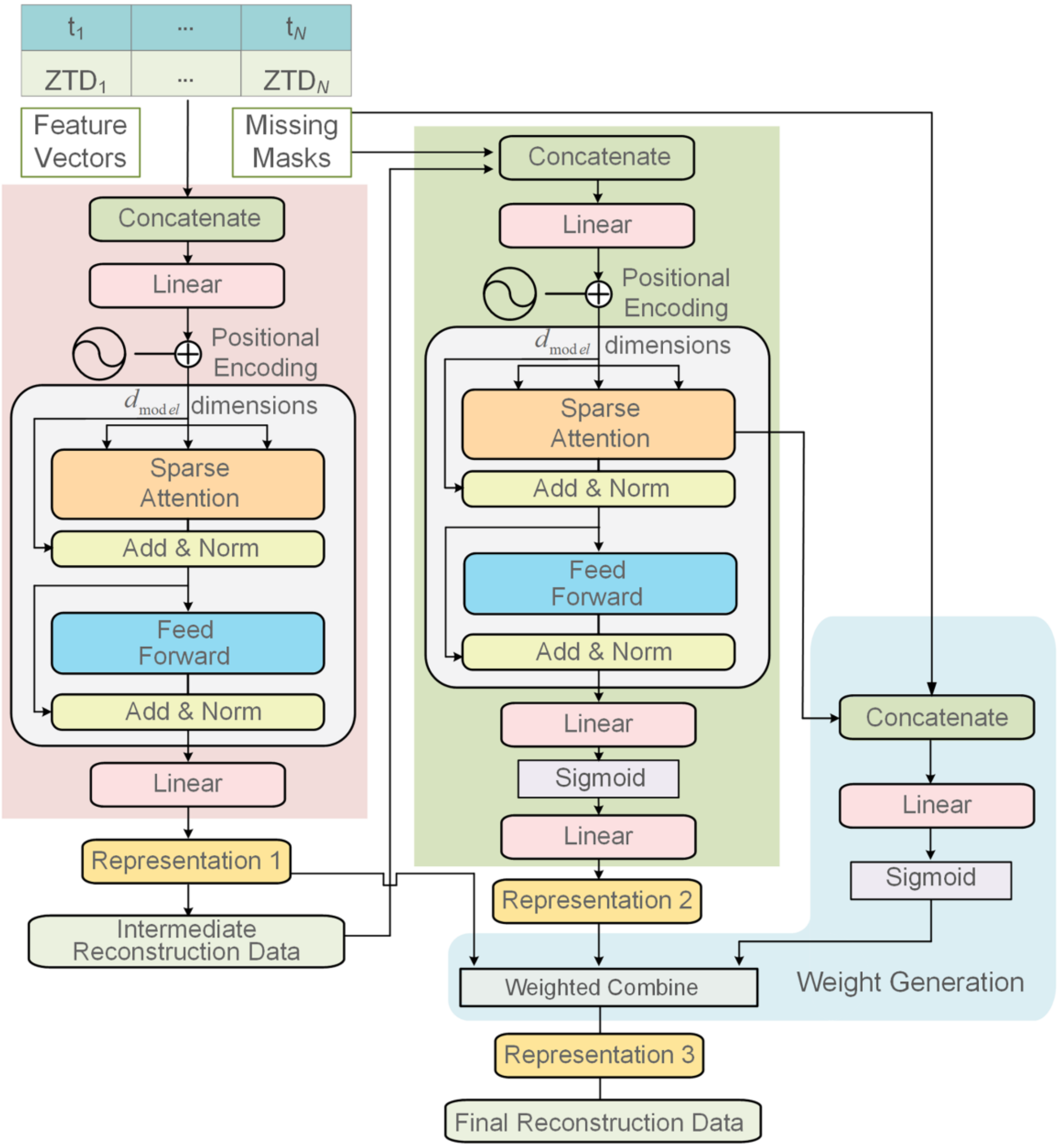

Figure S3. Diagram of the SASR Model Structure for Data Reconstruction, related to STAR Methods. The pink and light green regions represent the sparse self-attention modules, while the light blue region represents the weighted combination module.

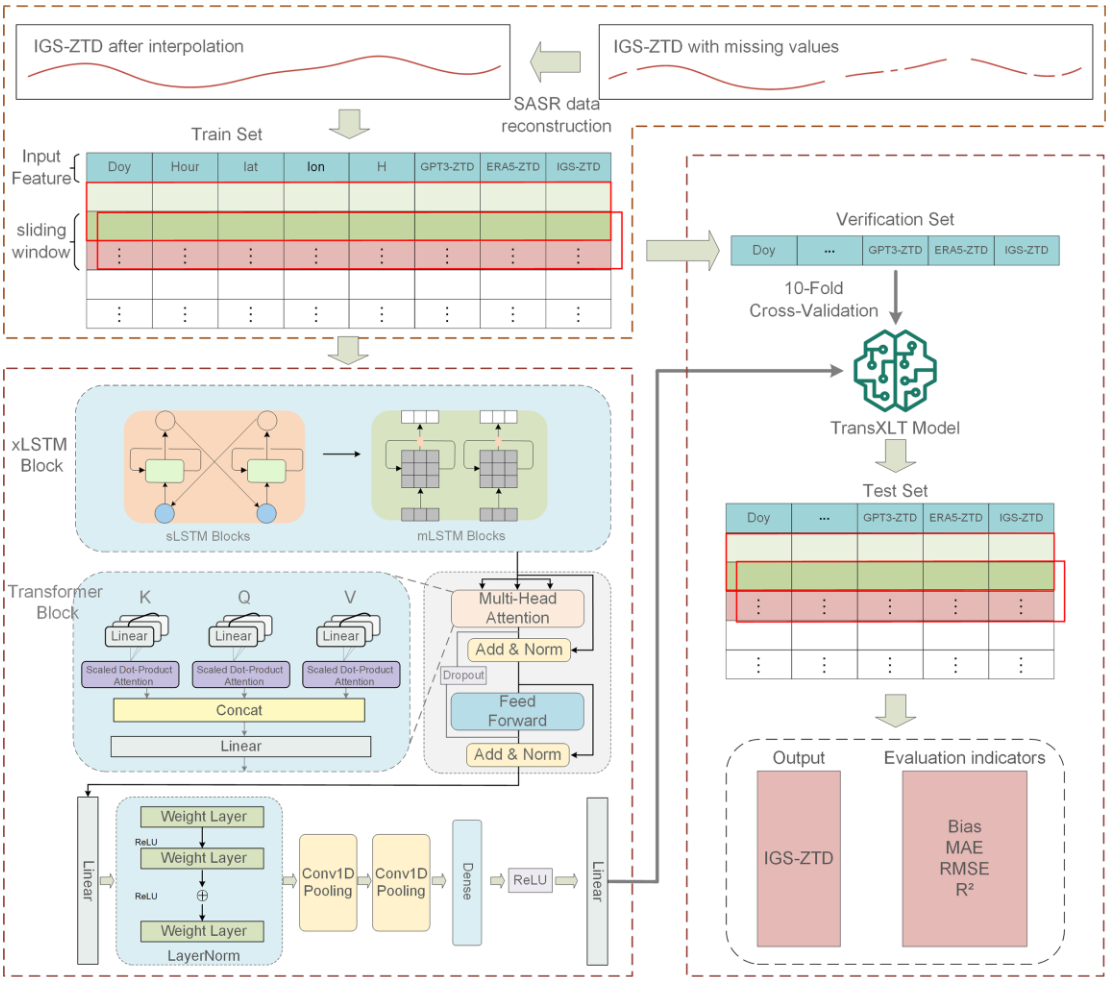

Figure S4. Flowchart of the TransXLT model for ZTD prediction, related to STAR Methods. The prediction process consists of three main stages: data extraction and preprocessing, model construction and training, and model testing and accuracy evaluation.
